# Cost-effectiveness of Legacy for Children™ for Reducing Behavioral Problems and Risk for ADHD among Children Living in Poverty

**DOI:** 10.4172/2375-4494.1000240

**Published:** 2015

**Authors:** Phaedra S Corso, Susanna N Visser, Justin B Ingels, Ruth Perou

**Affiliations:** Department of Health Policy and Management, University of Georgia, Wright Hall Office 315, 100 Foster Road, Athens, GA 30602, USA

**Keywords:** Economic evaluation, Cost-effectiveness analysis, Programmatic cost analysis, Behavioral problems, Attention-deficit/hyperactivity disorder

## Abstract

This paper describes the programmatic costs required for implementation of the Legacy for Children™ (*Legacy*) program at two sites (Miami and Los Angeles) and enumerate the cost-effectiveness of the program. *Legacy* provided group-based parenting intervention for mothers and children living in poverty. This cost-effectiveness analysis included two behavioral outcomes, behavioral problems, and attention-deficit/hyperactivity disorder (ADHD), and programmatic costs collected prospectively (2008 US$). Incremental costs, effects, the incremental cost-effectiveness ratio (ICER), and cost-effectiveness acceptability curves were estimated for the intervention groups relative to a comparison group with a 5 year analytic horizon. The intervention costs per family for Miami and Los Angeles were $16,900 and $14,100, respectively. For behavioral problems, the incremental effects were marginally significant (p=0.11) for Miami with an ICER of $178,000 per child at high risk for severe behavioral problems avoided. For ADHD, the incremental effects were significant (p=0.03) for Los Angeles with an ICER of $91,100 per child at high risk for ADHD avoided. *Legacy* was related to improvements in behavioral outcomes within two community-drawn sites and the costs and effects are reasonable considering the associated economic costs.

## Introduction

For the more than 15 million children living in poverty in the United States, there is an increased risk for poor health and developmental outcomes [1–8]. Poverty is associated with developmental delays, special education placement, and academic failures [9–13], along with poor health outcomes [1,14–18]. Furthermore, a strong link has been established between poverty and increased mental health issues [19–22] which are exacerbated by persistent poverty [22–24]. These associations continue as children become adults [16,25–29], including a reduction of 6 to 7 years in life expectancy [30]. Cutler and Richardson [31] estimated that an individual’s health capital over their lifetime is reduced by $124,000 (1990 US$) when raised in poverty, and others have estimated that $149 billion (2007 US$) in health capital is lost per year in the United States due to poverty [32].

Even more staggering are the family-level, healthcare-related, and crime-related economic impacts of specific disorders that are more common among children in poverty, such as attention-deficit/hyperactivity disorder (ADHD) [2,33,34]. ADHD prevalence is disproportionately high among children living in poverty [2] and is associated with impaired educational performance, delinquency, and increased use of school-based services [33]. Children and adolescents with ADHD cost have associated costs that are $38 to $72 billion more than others, primarily through increased health care and education costs [34]. In adulthood, ADHD is associated with increased absenteeism [35]; productivity losses of $87 billion to $138 billion (2010 US$) in the United States [34]; and higher rates of incarceration, psychiatric disorders, and death [36].

Recent research indicates the strong relationship between adverse experiences among low-income children and families and poor child development and health outcomes [37–41]. Research also documents the mitigating effects of early prevention and intervention programs on the relationship between the adverse experience of poverty and child development and health outcomes [42–45]. However, it is less clear how much these interventions cost or how the costs of these interventions compare to their outcomes. For example, in the Elmira Prenatal/Early Infancy Project (PEIP), families receiving home visits were found to save the government $1772 (1980 US$) per family compared to a randomized control group, with low-income families saving the government $3498 per family [44]. When program costs in this study were compared to savings in government expenditures, such as food stamps and Medicaid, and tax revenue from maternal employment, the savings was $180 per family. In the Chicago Child-Parent Centers (CPC) program, a benefit-cost analysis found a return to society of $7.14 (1998 US$) for every dollar invested in the program by improving economic well-being, increasing tax revenues, and decreasing government expenditures in education and crime [45].

The purpose of this research is to conduct a cost-effectiveness analysis of the Legacy for Children™ program, which was developed as a public health strategy to improve child health and development [46]. The program was developed using federal funds and resides within the public domain. Using data from a recent evaluation of *Legacy* [47], we describe the approach for collecting and analyzing the programmatic costs and we determine the cost-effectiveness of the program to reduce referable levels of behavioral concerns and risk for ADHD among participant children. This research is an important step towards the dissemination of an evidence-based public health intervention aimed at improving the developmental health of children born into poverty [48].

## Methods

### The legacy intervention

The Centers for Disease Control and Prevention (CDC) developed the *Legacy* program in collaboration with the University of California - Los Angeles (UCLA) and the University of Miami (UM) to focus on preventing the negative consequences of poverty on children. Perou et al. [46] previously described the methods and sample characteristics. The primary focus of the intervention is to provide a supportive, group environment that fosters self-efficacy and a sense of community, while providing developmentally appropriate information about child development. The anticipated outcome of the group intervention is improved quality of interaction between participating mothers and their children, which should serve to promote developmental outcomes. *Legacy* provides a unique approach compared to other early childhood interventions as it focuses on developing self-efficacy and a sense of community among mothers, rather than providing case management for the mother or child. *Legacy* has undergone testing of its effectiveness at two sites, Miami and Los Angeles (LA). In Miami, 300 participants were recruited in the hospital shortly after the child’s birth and randomized to either intervention or comparison groups; in LA 306 participants were recruited and randomized prenatally. Inclusion criteria included Medicaid-eligibility, living within the servable catchment area, having had some prenatal care, and being conversant in English.

Each site used the same intervention model (core components and goals), while developing a site-specific curriculum to fit their population’s needs. Intervention specialists who were trained in the intervention goals and delivery facilitated the sessions. At both sites, the curriculum included a segment each week on a topic of relevance to mothers with a child of a certain age. The intervention specialists also allowed time for unstructured discussion among the group members to build a sense of community among the mothers, and time each week for facilitated parent-child interaction. In Miami, mothers were invited to meet weekly for 1.5-hour sessions from a few weeks after birth until the time their child was 5-years of age. In LA, the structure of the program incorporated five 1-hour prenatal sessions followed by nine blocks of ten 1.5-hour sessions between birth and the child reaching 3 years of age. The group sessions alternated between mother-only sessions and sessions when the mother and child attended.

The Institutional Review Boards conducted human subject reviews at the CDC, Research Triangle Institute, UCLA, UM, and at Western IRB between 2005 and 2008 when UM contracted with them to conduct human subjects protection reviews.

### Effects

Programmatic effects and costs were prospectively assessed for N=381 (N=194 in Miami and N=187 in LA) mother-child dyads that participated in the *Legacy* trial and were followed-up through 5 years of age. A complete description of the *Legacy* intervention design [46] and results of the evaluation of socio-emotional and behavioral impacts [47] are reported elsewhere. The two main outcome measures for this analysis are risk for severe behavior problems and for ADHD. Statistical significance was assessed for both outcomes using the large-sample Wald test statistic [49]. Effects were considered significant when *p* ≤ 0.05 and marginally significant when p ≤ 0.1.

Behavioral problems were defined by the Devereux Early Childhood Assessment (DECA), a parent-reported rating scale (Cronbach’s alpha with parent raters of 0.71), which measures problem behaviors [50]. Although 1 standard deviation (SD) beyond the mean has been shown to result in a 71% correct classification [51], for the original program evaluation [47] and for this analysis we selected a cut-point of 2 SDs beyond the mean, which reflects criteria for referral to community assessment and services. The Strengths and Difficulties Questionnaire, hyperactivity-inattention subscale (SDQ-HI) was used to define risk for ADHD [52]. A recent report by Ullebø et al. [53] provided evidence for a cut-off value on the SDQ-HI (cut-off = 5) with good psychometrics for predicting ADHD (sensitivity = 79%, specificity = 86%). Finally, the dichotomous variables we created for effects were given a value of 1 for a DECA ≤ 69 (no severe behavioral problems) or for an SDQ-HI<5 (lack of hyperactivity-inattention) and values of 0 otherwise.

### Programmatic costs

Prospective collection of the programmatic costs required to implement and run *Legacy* took place during the period of October 2000 to March 2007 for the LA site and April 2001 to October 2008 for the Miami site. While intervention planning began before these dates, most of those early resource expenditures were not intervention-related but rather directed to building research capacity. However, it is possible that some pre-implementation costs were not included. In a few cases, these records contained errors and were supplemented by retrospective administrative records. Programmatic costs were assessed from the provider perspective, costs that accrued to the intervention provider, exclusive of participant or other non-provider costs, and included only the value of intervention delivery resources. All program costs were categorized based on two major activities: pre-implementation and implementation. Pre-implementation activities included recruitment, training, and other activities necessary initially to implement the intervention but excluding activities that are not part of the ongoing operations of the intervention. Implementation activities included group meetings, intervention administration, one-on-one contacts, and health consultations. Costs classified as research were excluded.

Data sources included site invoices and time diaries completed by the program providers, and information collected from site coordinators. The funding agency collected site invoices monthly, which allowed for the breakdown of costs into labor and non-labor cost categories. The site invoices included both the salary and fringe benefits paid to study personnel and the time diaries provided the allocation of these costs to non-research activities. Staff members who routinely performed two or more activities completed these diaries approximately twice a year documenting their time and activities conducted during the period of data collection. This information was used to estimate staff member’s percent time for non-research activities. Additionally, the researchers conducted retrospective interviews with study coordinators and site principal investigators (PIs) to complete gaps from site invoices and diaries. Finally, weekly summary reports included information about the number of active groups, the number of times that groups met, and participant attendance by month for both sites. Further details regarding the allocation of programmatic costs are provided in the Supplementary Information.

All final cost estimates are provided in 2008 US dollars (US$). The consumer price index for all household goods and services was used to adjust data from years prior to 2008 and to account for price inflation during the course of the intervention [54]. A 3% discount rate was used, in keeping with current recommendations, to adjust costs to present value [55].

### Cost-effectiveness analysis

We calculated incremental cost-effectiveness ratios (ICER) by separately comparing the *Legacy* program implemented in each site to the comparison scenario when effects were at least marginally significant. For this study, the ICER compares the difference in costs of *Legacy* and the control group (assuming costs for the control group were zero) to the difference in effects of these two groups. The interpretation of the ratio is the additional cost needed to produce a one percent reduction in the outcome and a smaller ICER implies a lower cost to achieve an outcome. Families randomly enrolled in the comparison arm of the study received the same developmental assessments of the intervention group, but they did not receive the core components of *Legacy*. The comparison families received any services that they normally would have received in usual care scenarios, such as community services for which they were eligible (e.g., primary health care services or services through early intervention programs). Families in *Legacy*, regardless of the study arm, were referred for further assessment and intervention if the child scored in the risk range on standardized assessments. As such, the comparison group was considered a mild intervention group.

For this analysis, we calculated separate ICERs for assessing program costs relative to severe behavior problems and high risk for ADHD. The intervention timeframe for assessing program costs is 8 years for both Miami (2001–2008) and for LA (2000–2007), which reflects the period of intervention at each of the two sites. However, a family was active for a maximum of 5 years in Miami and 3 years in LA, the longer timeframe of the intervention reflects the rolling nature of enrollment. The analytic horizon, the time from the start of the intervention to the latest assessment of costs and effects, for the analysis is 5 years as the two main effects are more accurate at older ages and provide an indication of school readiness.

Only families with a 5-year follow-up assessment were included when both mean effects and costs were estimated. For each effect, families were coded based on a “successful” 5-year assessment, where the effect (π) was coded as a 1 when the child tested in the normal range (see discussion above for more details). Calculation of the following cost-effectiveness parameters is based on prior work [56]. The probability of a successful 5-year assessment by site, intervention arm, and effect, was estimated as the sum of π_i_ divided by the number of families in either the treatment (n_T_) or comparison group (n_C_). The incremental difference in effects is then the difference in these two proportions. The mean total intervention costs $\left({\bar{c}}_{r}\right)$ for each site was estimated as the sum of the total intervention costs for each family (C_Ti_) divided by (n_T_). Since the total costs for the comparison group were zero, the incremental mean total costs (Δ_*C*_) for each site is equal to$\left({\bar{c}}_{r}\right)$.

The ICER is estimated by Δ_*C*_/Δ_*E*_ when the effect is at least marginally significant for a site. Due to the difficulty in interpretation of variability around ICER estimates, we also include an analysis of the incremental net benefit (INB) of *Legacy* for each effect at each site. The INB converts the outcome into monetary units, based on a specific monetary value given to each unit of outcome. The INB is estimated by *b*_λ_ = Δ_*E*_λ − Δ_*C*_ where λ represents the willingness to pay threshold value for the effect of interest. In this study, threshold values correspond to the amount a decision maker would be willing to pay to prevent either a referral for a behavioral problem or a case of ADHD. A positive INB suggests that *Legacy* is cost-effective for a specific effect at a given value of λ. The cost-effectiveness acceptability curves (CEACs), a means to illustrate the uncertainty around the parameter estimates by plotting the probability that an intervention is cost-effective at different threshold values, are created based on the INB at different threshold values [57–59]. A decision maker has the option to select a threshold value that he or she deems appropriate and judge the likelihood that *Legacy* is cost-effective for an outcome of interest. Full methodological details including equations for the variance of estimates and for the construction of the CEACs are provided in the Supplementary Information.

Four different assumptions are tested in a series of one-way sensitivity analyses, three impacting costs and the other effects. First, the discount rate is varied between 0% (no discounting of costs) and 5%. Second, the mean total intervention costs were based on all families, 180 for Miami and 189 for LA. Third, in the base case all of the pre-implementation costs were allocated to the families in the intervention, which assumes that no further families would receive the intervention. For this sensitivity analysis, conditions are considered where only half or none of these costs are allocated to families, the latter estimating intervention costs based only on implementation activities. Finally, the missing effects at the 5-year assessment are imputed using results from the four-year assessment, if available. In this case, the sample sizes are 218 and 215 for Miami and LA, respectively.

## Results

At 5 years follow-up, 194 mothers in Miami and 187 mothers in LA completed the DECA and SDQ-HI and are included in this analysis. At baseline, these mothers were on average 24.0 years of age, non-Hispanic black (68.5%) or Hispanic (27.5%), and all had incomes below 200% of the federal poverty level. Mothers at the two sites differed significantly only in ethnicity; while most in Miami were non-Hispanic black (90.2%), LA consisted of roughly equal proportions of non-Hispanic black and Hispanic mothers.

### Effects

For behavioral concerns, incremental effects were only marginally significant in Miami (p=0.110) at 5 years follow-up, and not significant in LA (p=0.267), showing that 9.5% of families in Miami reported severe behavioral problems relative to comparison families [47]. For ADHD risk, incremental effects were not significant in Miami (p=0.241), but were statistically significant in LA (p=0.028), showing that 15.5% fewer *Legacy* families in LA reported children at high risk for ADHD, relative to comparison families [47].

### Programmatic costs

The appendix provides a breakdown of implementation costs by category at both sites. For the cost-effectiveness analysis, pre-implementation costs were combined with these implementation costs and discounted by 3%. In Miami, the average *Legacy* costs per family were $16,900 and in LA were $14,100.

### Cost-effectiveness analysis

Table 1 presents the average per family costs of *Legacy*, the percentage of children likely to have severe behavioral problems (effects), incremental costs, incremental effects, and ICERs if differences in effects were found to be significant. Only in Miami were effects found to be marginally significant, and therefore the ICER is $178,000 per child at high risk for severe behavioral problems avoided for *Legacy* families compared to comparison families.

Table 2 presents the average per family costs of *Legacy*, the percentage of children at high risk for ADHD (effects), incremental costs, incremental effects, and ICERs if differences in effects were found to be significant. Effects in LA were found to be significant, and therefore the ICER is $91,100 per child at high risk for ADHD avoided, comparing *Legacy* families to comparison families.

### Sensitivity analyses

**Figure 1:**
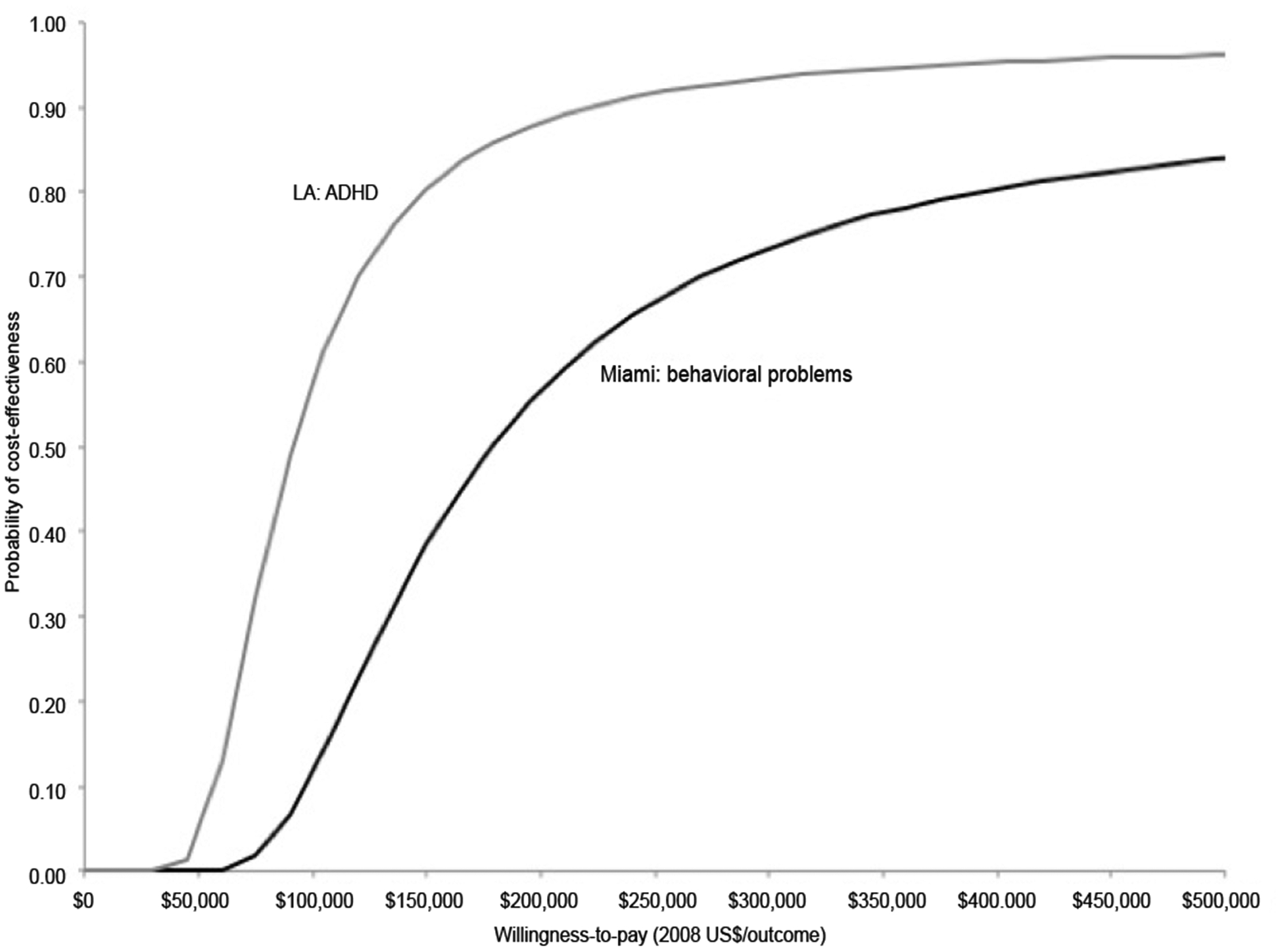
Cost-effectiveness acceptability curves for a child with severe behavioral problems avoided in Miami and a child at high risk for ADHD avoided in LA.

Figure 1 presents the CEACs for severe behavioral problems in Miami and high risk for ADHD in LA with the probability that *Legacy* was cost-effective, plotted from a willingness to pay of $0 to $500,000. There is greater than a 50% probability of cost-effectiveness by $100,000 in Miami and $200,000 in LA. Therefore, if a decision maker’s threshold is greater than $100,000, there is a greater than 50% probability that *Legacy* was cost-effective in Miami for reducing severe behavioral problems. Similarly, if a decision maker’s threshold is greater than $200,000, there is a greater than 50% probability that *Legacy* was cost-effective in LA for avoiding a high risk of ADHD. Table 3 reports ICERs for one-way sensitivity analyses around several key assumptions. For severe behavioral problems in Miami, the ICERs range from $140,000 to $194,000 and for ADHD risk in LA, from $70,200 to $95,100. It is unlikely that any of these assumptions would lead to major changes in the interpretation of study results.

## Discussion

In Miami, we found that programmatic cost per child who would have had a severe behavioral problem without *Legacy* was less than $200,000. In LA, we found that the programmatic cost per child who would have been at high risk for ADHD without *Legacy* was less than $100,000. However, while the sensitivity and specificity for the SDQHI are high, the positive predictive value is only 24% [53], which means that only one in four of these individuals would be expected to eventually receive an ADHD diagnosis. Therefore, the cost per child for ADHD avoided may be closer to $400,000. One-way sensitivity analyses of analysis assumptions did not significantly impact the interpretation of the study results.

While typical willingness to pay thresholds for severe behavioral concerns and ADHD are not known, the magnitude of these values are influenced by the resources required to treat or manage these concerns. For example, the cross-sector economic impact of ADHD has been estimated between $13,235 and $19,246 (2008 US$) per person per year [59]. Other research has shown that the health care costs of children with ADHD are between $573 to $1531 (2008 US$) per year more than children without ADHD [60]. Identifying programs that have a positive impact on the behavioral health of children in poverty could have a substantial impact on the estimated $21-$44 billion in health care-associated costs and $15-$25 billion in education costs associated with ADHD [34].

An important feature and strength of this analysis and how it differs from what has been published previously including the use of primarily prospective cost data collection to capture the resources used for the *Legacy* intervention at two diverse sites. Most other published analyses of early childhood interventions rely on retrospective analysis, where the researchers reconstruct costs based on assumptions about the program staff and other resources required to deliver the intervention. Poor record keeping and study design flaws often plague primarily retrospective analyses, which can bias the results and lead to questionable validity [61].

Although both sites demonstrated effects for behavioral problems, these effects were demonstrated on different behavioral assessment measures and at different assessment time-points, making it difficult to generalize the protective effects, and the associated costs, to other communities. Further, only marginal significance was achieved for the behavioral problems outcomes in Miami and no significance for the behavioral problems outcome in LA and ADHD outcome in Miami. Poverty is heterogeneous, resulting in demographic site differences across the two sites. These demographic differences as well as *Legacy* implementation differences (e.g., periodicity of the meetings) could have contributed to the differences in the effects seen in the *Legacy* evaluation. Nevertheless, this analysis suggests that there are protective effects of *Legacy* on behavioral outcomes and the costs associated with this effort equates to approximately $100,000-$200,000 per child at high risk avoided. Future assessments of the original *Legacy* participants should serve to further inform the long-term impacts demonstrated by *Legacy*. Another limitation is that participants were not surveyed regarding their time spent on the intervention outside of regular group meetings. For example, groups may have met outside of *Legacy*. This limits the ability for estimating opportunity costs and eventually estimating costs at the societal perspective. Despite these limitations, the results of this cost-effectiveness study suggest that *Legacy* was related to improvements in behavioral outcomes among children in poverty. The costs associated with these effects are reasonable when considering the considerable economic costs associated with significant behavioral concerns and ADHD.

### CDC Disclaimer

The findings and conclusions in this report are those of the authors and do not necessarily represent the official position of the Centers for Disease Control and Prevention or the Health Resources and Services Administration.

## Figures and Tables

**Table 1:** Costs, effects, and cost effectiveness of the Legacy for Children™ program in reducing the likelihood of children requiring a referral for severe behavioral problems.

	Costs	Effects^1^	Incremental Costs	Incremental Effects	ICER^2^
Miami (N=194)					
Control	$0	0.740			
Intervention	$16,900	0.835	$16,900	0.095(p=0.11)	$178,000
Los Angeles (N=187)					
Control	$0	0.831			
Intervention	$14,100	0.888	$14,100	0.057(p=0.27)	N/A

1 Proportion of children testing in the normal range, not requiring a referral for severe behavioral problems.

2 ICER is in units of 2008 US$ per child at high risk for severe behavioral problems in the Legacy group, relative to the comparison group.

**Table 2:** Costs, effects, and cost effectiveness of the Legacy for Children™ program in reducing the likelihood of children being at high risk for ADHD.

	Costs	Effects^1^	Incremental Costs	Incremental Effects	ICER^2^
Miami (N=194)					
Control	$0	0.5342			
Intervention	$16,900	0.6198	$16,900	0.086 (p=0.24)	N/A
Los Angeles (N=187)					
Control	$0	0.5775			
Intervention	$14,100	0.7328	$14,100	0.155 (p=0.03)	$91,100

1 Proportion of children testing in the normal range, not at high risk for ADHD.

2 ICER is in units of 2008 US$ per child at high risk for ADHD avoided in the Legacy group, relative to the comparison group.

**Table 3:** Results of each one-way sensitivity analysis for behavioral problems outcome in Miami and ADHD outcome in LA.

Parameter			Miami:behavioral problems	LA:ADHD
Base			$178,000	$91,100
Discount rate				
	0%		$194,000	$95,100
	5%		$169,000	$88,700
All families included for costs^1^			$140,000	$70,200
Partial pre-implementation costs				
		0%	$168,000	$69,300
		50%	$173,000	$80,300
Impute using year 4 effects			$177,000	N/A^2^

1 The per family total intervention costs for Miami are $13,300 and for LA are $10,900.

2 Effects not significant, p=0.20
